# Inadvertent extravasations of norepinephrine

**DOI:** 10.1002/ccr3.6516

**Published:** 2022-10-22

**Authors:** Ravi Ranjan Pradhan

**Affiliations:** ^1^ Department of Internal Medicine Madesh Institute of Health Sciences Janakpurdham Nepal

**Keywords:** bulla, debridement, inadvertent extravasations, norepinephrine, tissue necrosis

## Abstract

A male patient of diabetic ketoacidosis and septic shock was started on norepinephrine infusion following which he developed bulla and subcutaneous tissue ischemia in the event of inadvertent extravasations of norepinephrine. The patient improved after management with mechanical debridement of necrosed tissue and regular dressing of the wound. The use of higher concentration of norepinephrine via peripheral intravenous route may lead to vasoconstriction and subcutaneous tissue ischemia due to inadvertent extravasations.

## 1 DESCRIPTION

**FIGURE 1:**
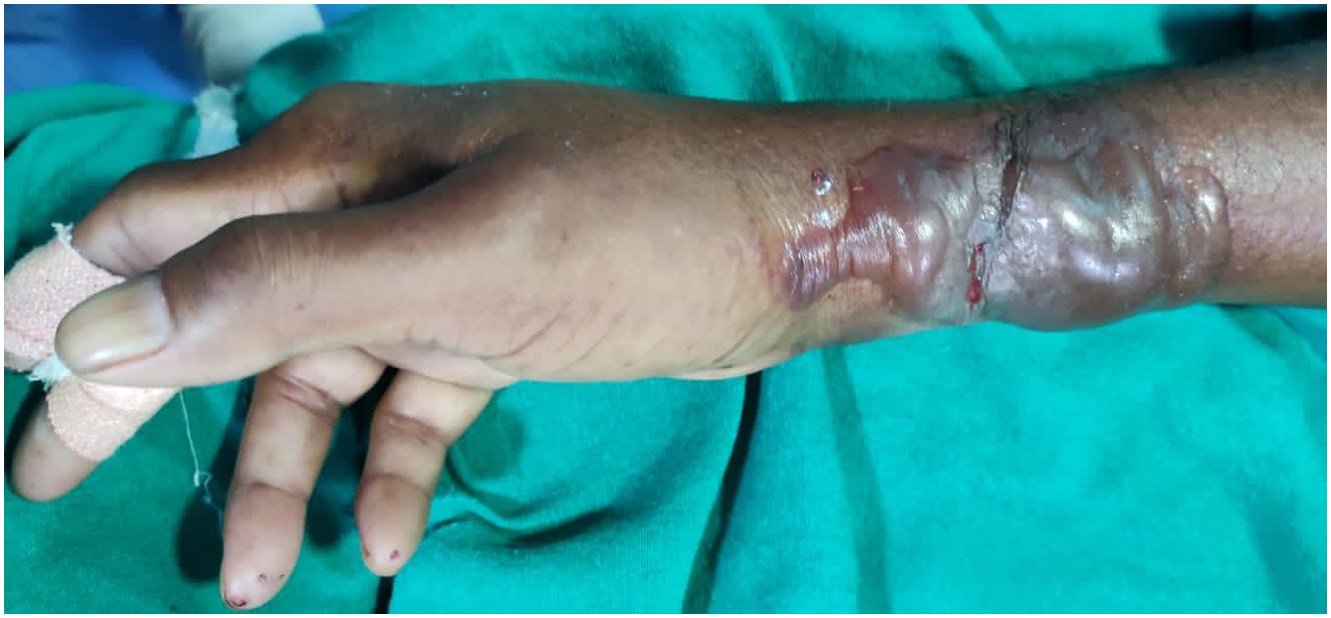
Bulla on right forearm around the peripheral intravenous site

A 63‐year‐old male patient presented in emergency with diabetic ketoacidosis and septic shock. He was started on insulin infusion, fluid resuscitation, and antibiotic. The correct positioning of peripheral intravenous (IV) cannula was assessed by flushing with 10 ml of normal saline. Owing to the perceived urgency, norepinephrine infusion (10 mcg/min) was initiated for hemodynamic support through a peripheral intravenous (PIV) line. Following hemodynamic stability after 48 h of norepinephrine PIV infusion, patient developed a large bulla on his right forearm around the peripheral intravenous site (Figure 1). A diagnosis of subcutaneous tissue ischemia in the event of inadvertent extravasations of norepinephrine was made. Norepinephrine infusion was discontinued immediately. The patient was treated with mechanical debridement of necrosed tissue followed by regular dressing of the wound.

In a study conducted by Pancaro et al., out of 14,385 patients receiving general anesthesia as well as PIV norepinephrine while undergoing surgery, five (0.035%) had an extravasation event, for an estimated risk of one to eight events per 10,000 patients. The norepinephrine infusions that extravasated were in a dose range of 0.02 to 0.05 mcg/kg/min (median administered dose was 40 mcg).^1^ In another study of Lewis et al (*n* = 202), the extravasation rate was 4% (median administered dose was 0.08 mcg/kg/min).^2^

The pathophysiology involves relatively higher concentration of norepinephrine in both the recipient vein and adjacent blood vessels following extravasations that lead to vasoconstriction and increased vascular permeability.^3^ The treatment of extravasation injuries includes prompt discontinuation of the peripheral intravenous infusion, administration of phentolamine (ɑ‐adrenergic antagonist), and debridement of necrosed tissue.^1^, ^2^

It is important to grade the severity of extravasation for patient's management. It is graded as the following: grade 1 (intact skin), grade 2 (blanched skin, erythema), grade 3 (necrosis/ulceration causing severe tissue damage warranting surgical intervention), grade 4 (life‐threatening warranting immediate intervention), and grade 5 (death).^1^ One should develop a detailed protocol for administering norepinephrine through a PIV line, which includes use of a vein >4 mm on ultrasound, IV cannula size of 20 or 18 Guage, no hand/wrist/antecubital fossa positions, assessment of peripheral IV access every 2 h by nursing, and a maximum duration of 72 h for peripheral IV use.^4^

## AUTHORS CONTRIBUTION

RRP conceived the study, collected data, and wrote the manuscript. RRP read and approved the final manuscript.

## FUNDING INFORMATION

None.

## CONFLICT OF INTEREST

None.

## CONSENT

Written informed consent was obtained from the patient to publish this report in accordance with the journal's patient consent policy.

## Data Availability

Data sharing not applicable to this article as no datasets were generated or analyzed during the current study.
